# Effects of intermittent pneumatic compression device on the improvement of tissue oxygen saturation and fluid clearance at the compression site

**DOI:** 10.3389/fphys.2026.1725445

**Published:** 2026-02-12

**Authors:** Masashi Aoyagi, Takayuki Komatsu, Ishin Togashi, Kai Iriguchi, Masashi Nagao, Atsushi Kubota, Hidenori Izawa, Yuki Someya, Koichi Oshio, Yuji Takazawa

**Affiliations:** 1 Juntendo Administration for Sports, Health and Medical Sciences, Juntendo University, Tokyo, Japan; 2 Department of Sports Medicine, Juntendo University, Tokyo, Japan; 3 Graduate School of Health and Sports Science, Juntendo University, Chiba, Japan; 4 Graduate School of Medicine, Juntendo University, Tokyo, Japan; 5 Innovative Medical Technology Research and Development Center, Juntendo University, Tokyo, Japan; 6 Department of Radiology, Juntendo University, Tokyo, Japan

**Keywords:** magnetic resonance imaging, metabolic waste removal, microcirculation, muscle oxygenation, venous outflow

## Abstract

**Introduction:**

Intermittent pneumatic compression devices (IPCDs) facilitate post-exercise recovery by increasing tissue oxygen saturation (rSO_2_) and reducing intramuscular interstitial fluid. However, limited evidence exists on time-course changes of these physiological responses during the intervention. This study aimed to examine the effects of IPCD use on rSO_2_ and fluid content in the lower limbs.

**Methods:**

We enrolled 59 healthy adults (37 males, 22 females) who underwent a 30-min IPCD intervention on their right leg at a target pressure of 90 mmHg. The rSO_2_ at the posterior calf was measured using near-infrared spectroscopy at baseline and across four periods (period 1: 0.5–3.5 min; period 2: 10–13 min; period 3: 20–23 min; and period 4: 27–30 min). T2-weighted magnetic resonance imaging was conducted pre- and post-intervention to assess fluid clearance.

**Results:**

The rSO_2_ values increased significantly from baseline (75.7% ± 5.8%) across all time periods (period 1: 79.5% ± 4.7%; period 2: 80.5% ± 4.7%; period 3: 80.8% ± 4.7%; and period 4: 81.2% ± 4.8%; p < 0.001). The values in periods 2, 3, and 4 were significantly higher than those in period 1 (p < 0.001), with no significant differences observed among periods 2, 3, and 4. Moreover, the T2 values significantly decreased post-intervention (Pre: median, 39.3; Post: 37.9; p < 0.001).

**Conclusion:**

IPCD use improves muscle oxygen saturation and facilitates fluid clearance within 30 min. Although oxygenation effects occur early, a 30-min IPCD session provides both oxygenation and fluid reduction benefits.

## 1 Introduction

Intermittent pneumatic compression devices (IPCDs) cyclically inflate and deflate air chambers using compressed air to apply pressure to the limb muscles. This compression and decompression cycle can facilitate the movement of body fluids, including venous blood and interstitial fluid (Chen et al., 2001), thereby influencing blood flow and fluid metabolism. In medical settings, IPCDs are generally used at low pressures for longer durations to prevent deep vein thrombosis or manage lymphedema (Gardner et al., 1990; Stranks et al., 1992). In sports settings, they are typically used at higher pressures for shorter durations to promote recovery, reduce exercise-induced edema, and alleviate muscle fatigue (Artés et al., 2024; Heapy et al., 2018; Hoffman et al., 2016; Winke and Williamson, 2018).

Oxygen transport in skeletal muscle is mediated by hemoglobin, which exists in two forms: oxygenated (oxyhemoglobin) and deoxygenated (deoxyhemoglobin). Oxyhemoglobin is predominant in arterial blood, while deoxyhemoglobin is more common in venous blood. Regional oxygen saturation (rSO_2_), measured using near-infrared spectroscopy (NIRS), reflects the proportion of oxygenated hemoglobin relative to total hemoglobin and serves as an index of the balance between arterial inflow and venous outflow at the measurement site (Messere et al., 2017).

A previous study reported that IPCD-induced compression promotes venous outflow, resulting in a relative increase in oxyhemoglobin and a subsequent rise in rSO_2_ (Messere et al., 2017). Theoretically, an increase in rSO_2_ may enhance ATP resynthesis (Cao et al., 2021) and facilitate oxidative clearance of metabolic byproducts such as lactate and hydrogen ions, thereby contributing to post-exercise recovery (Hanson et al., 2013; Huang et al., 2025). IPCD use can also facilitate the clearance of excess interstitial fluid that accumulates after exercise (Winke and Williamson, 2018), which in turn may reduce intramuscular pressure, improve force production (Sleboda and Roberts, 2020), and promote the convective clearance of metabolites (Chen et al., 2001). Indeed, enhancement of limb blood flow during recovery has been shown to be largely correlated with the restoration of subsequent anaerobic performance (Borne et al., 2017). However, the current body of evidence regarding the efficacy of IPCDs in enhancing muscular recovery remains inconsistent. While some studies suggest benefits (Artés et al., 2024; Hoffman et al., 2016; Maia et al., 2024a; Winke and Williamson, 2018), others report negligible effects (Martin et al., 2015; Overmayer and Driller, 2018), highlighting the need for further physiological clarification.

To elicit these theoretical benefits, IPCDs are typically applied for 20–60 min (Maia et al., 2024b), although the duration of application varies depending on the manufacturer’s protocol. Currently, limited scientific evidence exists on time-course changes of these physiological responses during the intervention (Maia et al., 2024a). Monitoring changes in rSO_2_ and visualizing fluid clearance during IPCD may help clarify the underlying mechanisms and guide the optimization of treatment. Therefore, this study aimed to investigate the effects of IPCD use on rSO_2_ and fluid content in the lower limbs. Furthermore, to establish fundamental physiological responses, we also recruited healthy individuals.

## 2 Materials and methods

### 2.1 Participants

A total of 59 healthy adult volunteers (37 males and 22 females; mean age, 23.2 ± 5.5 years) participated in this study (Table 1). We excluded individuals with lower limb injuries or pain that could interfere with IPCD use; those with cardiovascular, dermatologic, neurologic, or metabolic diseases (including diabetes mellitus); and those with a history of deep vein thrombosis within the past 6 months due to the risk of developing pressure-induced injuries or thrombosis. Individuals with implanted metal devices, such as pacemakers, were also excluded because they could not safely undergo magnetic resonance imaging (MRI). In addition, participants with subcutaneous fat thickness greater than 2 cm in the lower leg, as measured on axial T2-weighted MRI, were excluded because oxygen saturation in the muscle could not be accurately measured under these conditions. Body weight and body mass index were not considered for the inclusion or exclusion criteria.

**TABLE 1:** Demographic data of the participants.

Sex	Number	Age [years]	Height [cm]	Weight [kg]	Body fat percentage [%]
Male	37	21.7 ± 2.5	172.1 ± 5.6	70.1 ± 11.7	15.7 ± 3.7
Female	22	25.8 ± 7.9	159.5 ± 5.5	54.5 ± 7.1	27.2 ± 6.3

Written informed consent was obtained from all participants. The Research Ethics Committee of the Faculty of Medicine, Juntendo University, approved the study protocol (E22-0417). This study protocol was registered in UMIN-CTR before the commencement of the experiments (UMIN000050567; registration date: 30 April 2023).

### 2.2 Procedure

This study was conducted as a single-arm trial without a control group. Participants first underwent baseline assessment and MRI, followed by a 5-min rest in the supine position. Subsequently, the IPCD intervention was applied to the right leg for 30 min. After the intervention, the participants underwent an MRI. As the IPCD could not be used in the MRI room, the participants received the intervention nearby and walked approximately 2 m before undergoing the MRI. Laboratory conditions were standardized at approximately 24 °C and 45% humidity throughout the study.

### 2.3 Intervention

**FIGURE 1:**
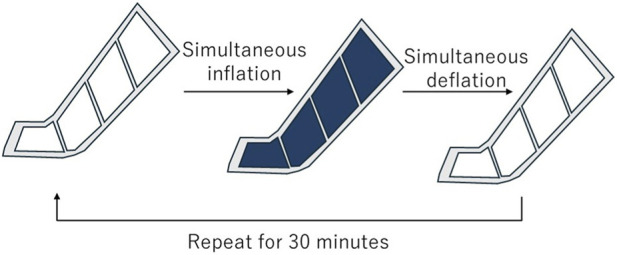
Schematic diagram of the intermittent pneumatic compression protocol. All four chambers were inflated simultaneously to the target pressure of 90 mmHg and then deflated simultaneously. This cycle was repeated for 30 min.

The participants received the IPCD intervention in the supine position using a Doctor Medomer DM-4S device (Nitto Kohki Co., Ltd., Japan). The leg sleeve, consisting of four air chambers, covered the entire leg from the toes to the thigh while leaving the toe tips exposed (Supplementary Figure S1). All chambers were inflated simultaneously for 20–30 s until the internal pressure monitored by the main unit of the device reached 90 mmHg (Figure 1). Once the pressure was achieved, the device automatically switched to decompression, which was defined as a single cycle. This inflation-deflation cycle was repeated continuously for 30 min.

### 2.4 Measurements

#### 2.4.1 Maximum lower leg circumference

The maximum circumference of the right lower leg was measured before and after IPCD intervention using a measuring tape. The measurement site was determined by visual estimation and confirmed by repeated measurements above and below this point.

#### 2.4.2 Subjective fatigue level in the leg

Subjective fatigue level for the right leg was rated on an 11-point numerical rating scale (0–10), where 0 = “no fatigue” and 10 = “fatigue as bad as you can imagine” (Wright et al., 2023). The participants rated their current fatigue immediately before and after the IPCD session; higher scores indicated greater fatigue.

#### 2.4.3 T2-weighted MRI

To assess tissue fluid content, T2-weighted imaging was performed using a 0.3-T MRI system (AIRIS Vento, FUJIFILM Medical Co., Ltd., Japan). The elevation or depression of the T2 values represented increasing or decreasing water content, respectively (Abdel-Aty et al., 2007). The axial image of the maximum circumference of the right lower leg, with a liquid-filled capsule affixed to the anterior surface as a landmark, was obtained immediately before and after the IPCD intervention. Each MRI scan required approximately 7 min. T2 values were calculated from the entire cross-sectional area, excluding fat tissue, using Gaussian distribution analysis (Takamori et al., 2019).

#### 2.4.4 rSO_2_

The rSO_2_ is widely used to evaluate tissue oxygen saturation in skeletal muscles (Rodríguez et al., 2015; Lubkowska et al., 2024) and allows real-time monitoring of tissue oxygenation when measured using NIRS. An increase in rSO_2_ indicates a relative increase in arterial blood within the muscle tissue.

The rSO_2_ value was calculated using the following formula:

$$\begin{array}{ll} & {rSO}_{2}=\frac{oxygenated\;hemoglobin\;}{total\;hemoglobin\;\left(=oxygenated+deoxygenated\;hemoglobin\right)}\; \end{array}$$

To indirectly evaluate changes in arterial and venous blood flow in the muscle tissue at the compression site, the rSO_2_ was continuously monitored from the start to the end of the intervention (Rodríguez et al., 2015). Measurements were conducted using the INVOS 5100C™ device (Covidien, Japan) with a sheet-type sensor (INVOS Soma Sensor™, Covidien, Japan) approximately 2 mm thick. The sensor was placed on the gastrocnemius muscle at the maximum circumference of the right leg (Lubkowska et al., 2024), corresponding to the location where T2 values were measured using MRI. The device measured the rSO_2_ approximately 2–3 cm beneath the skin (i.e., intra-gastrocnemius muscle in this study) and recorded data every 5–6 s throughout the 30-min IPCD intervention.

For analysis, the 30-min intervention was divided into baseline and four periods: 0.5–3.5 min (period 1), 10–13 min (period 2), 20–23 min (period 3), and 27–30 min (period 4) (Supplementary Figure S2). During each period, the IPCD underwent 4–5 cycles of compression and decompression. For each period, the maximum and minimum rSO_2_ values were defined by calculating the average maximum and minimum rSO_2_ values across the cycles.

#### 2.4.5 Interface pressures

Interface pressure was defined as the contact pressure between the IPCD and the skin. The pressures were measured on the right lower limb during IPCD intervention using a pressure measurement device (AMI 3037-10-SW-Ⅱ, AMI Techno Co., Ltd., Japan). Thereafter, thin sensors (approximately 1 mm thick) were placed at four sites: adjacent to the rSO_2_ sensor on the posterior lower leg, central anterior ankle, popliteal fossa, and medial thigh, where the major blood vessels are located. Following this, the interface pressure was recorded every second throughout the 30-min IPCD intervention. Peak pressure values were used in the analysis.

#### 2.4.6 Pulse wave detection

To evaluate the impact of IPCD on peripheral blood flow, pulse waves at the second digit foot were monitored using a pulse oximeter (Nellcor™ N-BSJ, Covidien, Japan). Measurements were recorded every 4 s. An absence of detection pulse waves for longer than 4 s was classified as a missed pulse.

#### 2.4.7 Adverse reactions

Any adverse reactions in the right lower leg related to the IPCD intervention, such as pain, numbness, or coldness, were monitored. The participants were asked about adverse reactions every 5 min during the IPCD intervention and instructed to report any reactions within 24 h following the intervention.

### 2.5 Statistical analysis

Normality of the data was assessed using the Shapiro–Wilk test. Changes in rSO_2_ within and across intervals were analyzed using paired t-tests and repeated-measures analysis of variance (ANOVA) with the Bonferroni correction. Pre- and post-intervention changes in T2 values, maximum lower leg circumference, and subjective fatigue level were evaluated using either the Wilcoxon signed-rank test or paired t-test, as appropriate. Differences in interface pressures between measurement sites were analyzed using repeated measures ANOVA with Bonferroni corrections. Additionally, regression analysis was performed to examine the relationship between interface pressures at all measurement sites with changes in rSO_2_ values (from baseline to maximum) and T2 values (from pre- to post-intervention). The unpaired t-test was also used to assess differences in the highest interface pressures between participants with and without missed pulse wave detection. To examine potential sex-based differences, between-sex comparisons were performed using analysis of covariance (ANCOVA). For rSO_2_, ANCOVA was conducted for each period-specific maximum rSO_2_ value with sex as a fixed factor and baseline rSO_2_ as a covariate. Similarly, for T2 values, leg maximal strength, maximum leg circumference, and subjective fatigue level, ANCOVA models were fitted using the pre-intervention value as a covariate and sex as a fixed factor. The interface pressures were compared between sexes using an unpaired t-test. The level of statistical significance was set at p < 0.05. All statistical analyses were conducted by M.A. and Y.S. as independent statisticians using SPSS version 20.0 (IBM Corp., Armonk, NY, United States). Because limited data were available for an *a priori* sample size calculation, a *post hoc* power analysis was performed for the primary outcome (rSO_2_) using G*Power 3.1.9.7 (Faul et al., 2009) based on the observed effect sizes.

## 3 Results

### 3.1 Change in rSO_2_

**FIGURE 2:**
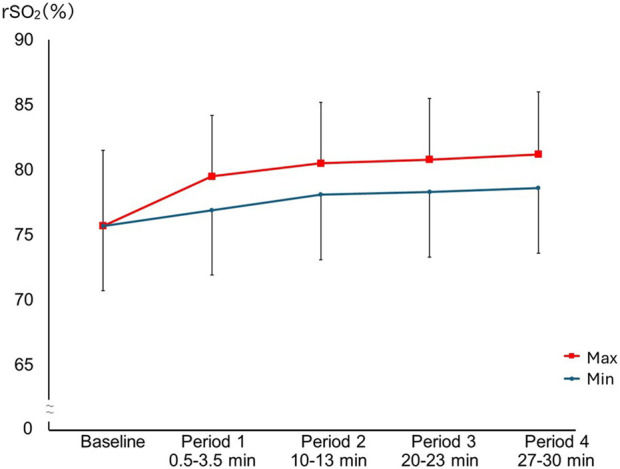
Changes in maximum and minimum rSO_2_ between each period. The maximum and minimum rSO_2_ values differed significantly across periods with [F(2.17, 125.58) = 111.71, p < 0.001, partial η^2^ = 0.66], and [F(2.16, 125.25) = 35.53, p < 0.001, partial η^2^ = 0.38, respectively]. Bonferroni-adjusted *post hoc* tests showed that both maximum and minimum rSO_2_ values were significantly higher in periods 1–4 than at baseline (p < 0.001), and were also higher in periods 2–4 than in period 1 (p < 0.001). No significant differences were observed among periods 2, 3, and 4 for either maximum or minimum rSO_2_. Squares and circles indicate mean maximum and minimum rSO_2_ values, respectively, and error bars indicate mean ± SD.

In each period, the rSO_2_ significantly increased from minimum to maximum (p < 0.001 for all periods) (Table 2). The maximum rSO_2_ values differed significantly across periods (*F*(2.17, 125.58) = 111.71, p < 0.001, partial η^2^ = 0.66). Bonferroni-adjusted *post hoc* comparisons showed that rSO_2_ values in all periods were significantly higher than baseline (baseline: 75.7% ± 5.8%; period 1: 79.5% ± 4.7%; period 2: 80.5% ± 4.7%; period 3: 80.8% ± 4.7%; period 4: 81.2% ± 4.8%; all p < 0.001). Similarly, the minimum rSO_2_ values differed significantly across periods (*F*(2.16, 125.25) = 35.53, p < 0.001, partial η^2^ = 0.38), and Bonferroni-adjusted comparisons showed that values in each period were significantly higher than those in baseline (period 1: 76.9% ± 4.9%; period 2: 78.1% ± 4.6%; period 3: 78.3% ± 4.8%; period 4: 78.6% ± 4.8%; all p < 0.001). Additionally, for both the maximum and minimum rSO_2_, values in periods 2, 3, and 4 were significantly higher than those in period 1 (Bonferroni-adjusted; all p < 0.001). However, no significant differences in values were observed among periods 2, 3, and 4 (Figure 2). Post hoc power analyses for the repeated-measures effect of period on maximum and minimum rSO_2_ indicated power > 0.99.

**TABLE 2:** Difference between the maximum and minimum rSO_2_ values within each period.

Period	Minimum rSO_2_ [%]	Maximum rSO_2_ [%]	p value	Effect size
Baseline	75.7 ± 5.8		-	-
1 (0.5–3.5 min)	76.9 ± 4.9	79.5 ± 4.7	p < 0.001	d = 2.57
2 (10–13 min)	78.1 ± 4.6	80.5 ± 4.7	p < 0.001	d = 2.35
3 (20–23 min)	78.3 ± 4.8	80.8 ± 4.7	p < 0.001	d = 2.09
4 (27–30 min)	78.6 ± 4.8	81.2 ± 4.8	p < 0.001	d = 2.07

Paired t-tests.

rSO_2_, regional oxygen saturation.

### 3.2 Changes in T2 values, maximum circumference, and subjective fatigue level

After excluding six participants because of incomplete or unclear MRI data, the T2 values significantly reduced after the IPCD intervention (pre: median 39.3, interquartile range (IQR) 37.8–40.6; post: 37.9, IQR 37.2–39.1; Z = −6.05, p < 0.001, r = 0.83) (Table 3). The maximum circumference of the right lower leg significantly decreased after IPCD intervention (pre: mean 36.7 ± 2.8 cm; post: mean 36.1 ± 2.8 cm; t(58) = 9.45, p < 0.001, Cohen’s d = 1.23) (Table 3). Similarly, the subjective fatigue levels significantly decreased (pre: median 2.0, IQR 0.5–3.0; post: median 2.0, IQR 0.0–2.0; Z = −3.44, p < 0.001, r = 0.45) (Table 3).

**TABLE 3:** Pre- and post-intervention changes following intermittent pneumatic compression.

	Pre intervention	Post intervention	p value	Effect size
T2 values	39.3 [37.8–40.6]	37.9 [37.2–39.1]	p < 0.001^a^	r = 0.83
Fatigue level	2.0 [0.5–3.0]	2.0 [0.0–2.0]	p < 0.001^a^	r = 0.45
Circumference of the leg [cm]	36.7 ± 2.8	36.1 ± 2.8	p < 0.001^b^	d = 1.23

Data are presented as mean ± standard deviation or median [interquartile range].

^a^ Wilcoxon signed-rank test.

^b^ Paired t-tests.

### 3.3 Interface pressures during IPCD intervention

**FIGURE 3:**
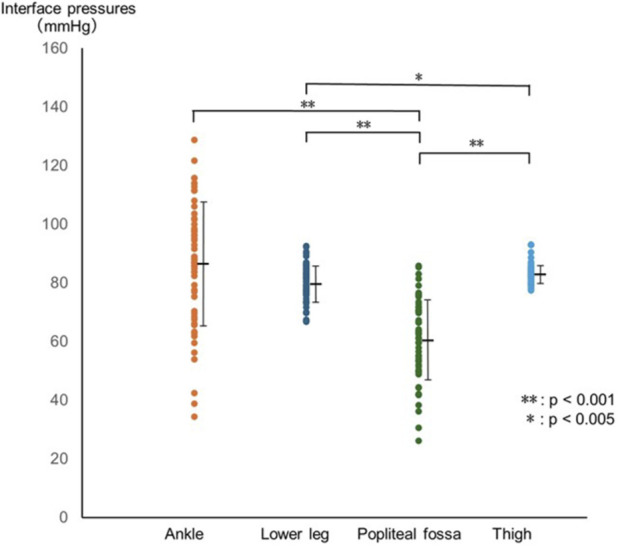
Differences in interface pressures between measurement sites. Interface pressures differed significantly across regions (F(1.71, 95.71) = 44.42, p < 0.001, partial η^2^ = 0.4). Bonferroni-adjusted *post hoc* tests indicated that pressures at the popliteal fossa were lower than those at the ankle (p < 0.001), lower leg (p < 0.001) and thigh (p < 0.001). Pressures at the lower leg were also lower than those at the thigh (p = 0.001). Circles represent individual values, and black bars indicate mean ± SD.

Interface pressures differed significantly across regions (F(1.71, 95.71) = 44.42, p < 0.001, partial η^2^ = 0.44). Bonferroni-adjusted *post hoc* comparisons showed that pressures at the popliteal fossa were significantly lower than those at the ankle, lower leg, and thigh (popliteal fossa: 60.9 ± 13.9 mmHg; ankle: 86.6 ± 21.0 mmHg; lower leg: 80.0 ± 6.1 mmHg; thigh: 83.3 ± 3.1 mmHg; all p < 0.001). In addition, pressures in the lower leg were significantly lower than those in the thigh (p = 0.001), whereas no significant differences were observed between the ankle and lower leg (p = 0.183) or between the ankle and thigh (p = 1.000) (Figure 3). The interface pressure exceeded 90 mmHg in 26 participants at the ankle, three at the lower leg, and three at the thigh. No significant associations were found between the interface pressures and changes in rSO_2_ values (ankle: p = 0.10; lower leg: p = 0.57; popliteal fossa: p = 0.34; thigh: p = 0.74) or T2 values (p = 0.93).

### 3.4 Pulse wave detections during IPCD intervention

In 32 out of 59 participants, pulse waves at the second digit of the foot were intermittently missed during the compression phase, with each episode lasting between 4 and 36 s. Comparison between participants with and without these missed pulse wave detections revealed no differences in peak interface pressures at any measurement site (Supplementary Table S1).

### 3.5 Adverse reactions

Two participants reported transient mild coldness in the toes of the right leg during IPCD intervention. Because these symptoms were tolerable and did not require discontinuation of the intervention, both participants completed the full protocol and were included in the final analyses. The symptoms resolved by the end of the intervention. Notably, no other adverse reactions were noted during or after the intervention.

### 3.6 Sex-based differences

There were significant differences between sexes in pre-intervention T2 values (Z = −5.16, p < 0.001, r = 0.71) and maximum leg circumference (*t*(57) = 3.69, p < 0.001, Cohen’s *d* = 0.99) (Table 4). After adjusting for baseline rSO_2_, there were significant sex differences in rSO_2_ changes during period 3 (F(1,56) = 9.67, p = 0.003, partial η^2^ = 0.15) and period 4 (F(1,56) = 5.99, p = 0.018, partial η^2^ = 0.10), with higher adjusted mean rSO_2_ in males (period 3: males 81.6% ± 0.4% vs. females 79.7% ± 0.5%; period 4: males 81.9% ± 0.5% vs. females 80.0% ± 0.6%). No significant differences were observed in period 1 (F(1, 56) = 0.85, p = 0.359, partial η^2^ = 0.02) or period 2 (F(1, 56) = 2.53, p = 0.117, partial η^2^ = 0.04). After adjustment for baseline values, no significant sex-based differences were observed in post-intervention T2 values (F(1, 50) = 2.38, p = 0.129, partial η^2^ = 0.05), maximum leg circumference (F(1, 56) = 0.34, p = 0.561, partial η^2^ = 0.01), or fatigue (F(1, 56) < 0.001, p = 0.996, partial η^2^ < 0.001). Regarding interface pressures, the mean pressure at the ankle was significantly higher in males than in females (males mean 92.9 ± 17.6 mmHg vs. females mean 75.8 ± 22.4 mmHg, p = 0.002, Cohen’s d = 0.88). No significant sex-based differences were observed in the other variables.

**TABLE 4:** Sex-based differences at the pre-intervention.

	Males (n = 37)	Females (n = 22)	p value	Effect size
rSO_2_ at baseline [%]	74.7 ± 5.9	77.3 ± 5.4	p = 0.104^a^	d = 0.44
T2 values	38.1 [37.2–39.3]	40.7 [40.4–43.2]	p < 0.001^b^	r = 0.71
Fatigue level	2.0 [0.0–3.5]	2.0 [0.8–3.3]	p = 0.614^b^	r = 0.07
Circumference of the leg [cm]	37.6 ± 2.7	35.1 ± 2.2	p < 0.001^a^	d = 0.99

Data are presented as mean ± standard deviation or median [interquartile range].

^a^ Unpaired t-test.

^b^ Mann–Whitney U test.

## 4 Discussion

This study investigated the effects of IPCD on muscle tissue by monitoring changes in rSO_2_ and fluid content. However, caution is required in interpreting the results because no control group was established. The substantial rSO_2_ changes observed in this study may be primarily attributable to the IPCD intervention as transient hemodynamic changes following the transition to the supine position can resolve rapidly (within approximately 10 s) (Toska and Walløe, 2002). In contrast, as fluid redistribution due to gravity occurs more gradually, the observed reductions in T2 values may reflect the combined effects of the IPCD intervention and supine positioning. Within this context, our findings revealed significant increases in rSO_2_ levels during the 30-min intervention, accompanied by reductions in T2 values and lower leg circumference. Continuous monitoring revealed that the rSO_2_ peaked 10 min after the onset of IPCD use. These findings suggest that IPCD in the supine position effectively enhances muscle oxygen saturation while facilitating localized fluid clearance.

During the 30-min intervention, the rSO_2_ values remained consistently higher than baseline, a pattern also observed in a previous study (Messere et al., 2017). Generally, an increase in rSO_2_ can result from increased arterial blood, reduced venous blood, or a combination of both. In this study, considering the significant reduction in T2 values, the increase in rSO_2_ was primarily caused by a reduction in deoxygenated venous blood. According to our findings, the rSO_2_ values plateaued after 10 min, and the T2 values decreased 30 min after initiation of the intervention. Although we did not evaluate the time-course of T2 changes during the intervention, these results suggest that 30 min of IPCD use in the supine position can achieve both improved oxygen saturation and fluid reduction.

Subjective fatigue levels also declined following the IPCD intervention. Our findings indicate that decreased T2 values and increased rSO_2_ may correspond to reduced subjective fatigue. These findings are consistent with those of previous studies, which suggest that the removal of excessive interstitial fluid and metabolic waste, as well as improved muscle oxygenation, can facilitate recovery from muscle fatigue (Born et al., 2013; Percival et al., 2022). Although the current participants were healthy individuals without experimentally induced fatigue, IPCD in the supine position facilitated the reduction of interstitial fluid. This may have reduced metabolic waste accumulated through daily activities and intratissue pressure, thereby decreasing nociceptive input and alleviating perceived fatigue (Amann et al., 2020; Wilke and Behringer, 2021). However, in this study, we could not determine the relative contributions of reduced T2 values versus increased rSO_2_ to subjective fatigue reduction.

The observed sex-based differences in rSO_2_ dynamics could be related to the disparity in interface pressures. Female participants exhibited significantly lower interface pressures at the ankle compared to males. During IPCD compression, if compression at the ankle is lower than other sites of the lower limb, venous blood may be less effectively propelled proximally, leading to peripheral venous pooling which may attenuate the increase in rSO_2_ observed in the latter phases of intervention. This lower ankle pressure in females is likely attributable to anatomical differences. Because the ankle region has minimal soft tissue covering bony prominences, the smaller limb circumference in females may have resulted in reduced sleeve conformity, leading to lower local pressure (Lurie et al., 2008; Zhao et al., 2019). It should be noted that, in this study, interface pressure was measured only on the anterior ankle; thus, the actual compression applied to other regions important for venous return, such as posterior to the medial malleolus, was not directly assessed. Nevertheless, adjusting the sleeve fit to match individual foot and ankle morphology may help equalize ankle pressure. Importantly, despite these differences in interface pressures and rSO_2_, no sex differences were observed in T2 value reduction or subjective fatigue relief. This indicates that the impact of lower ankle pressure on fluid dynamics and fatigue recovery was limited in this context and sex-based variations may not significantly impact on the overall therapeutic benefits (Artés et al., 2024). Collectively, this observation suggests that the physiological benefits of IPCD, particularly for localized fluid clearance, might be achieved at a pressure threshold lower than the targeted 90 mmHg, highlighting a potential area for future research into the ‘minimum effective pressure’ for optimizing recovery protocols.

Although the IPCD used in this study was designed to deliver a uniform internal pressure of 90 mmHg across the leg, the actual interface pressures varied among anatomical sites and between individuals. In some participants, the interface pressures at the ankle exceeded 90 mmHg, and toe pulse waves were transiently undetectable during IPCD use. As discussed, such variations are likely influenced by individual differences in limb morphology and tissue stiffness (Lurie et al., 2008; Zhao et al., 2019). Regarding the pulse wave loss, given that arterial occlusion typically requires pressures higher than 90 mmHg (Loenneke et al., 2012), these transient pulse absences are unlikely to indicate arterial blockage. In this study, neither elevated interface pressures nor missed pulse wave detection was associated with any adverse symptoms. Therefore, although variations in the local pressure and transient pulse loss may occur during IPCD use, they may not be of particular concern to healthy individuals.

While sequential or peristaltic compression patterns are widely used in sports settings, evidence regarding their superiority over simultaneous compression pattern for enhancing blood flow and tissue oxygenation remains conflicting (Nandwana and Ho, 2019; Yanaoka et al., 2022). Given these discrepancies, simultaneous compression pattern was adopted in the present study to apply uniform pressure across the lower leg. This pattern eliminated the confounding variable of inflation timing, providing a controlled condition suitable for clarifying the fundamental physiological responses to intermittent compression investigated in this study.

Considering the participants in this study, our findings support the potential utility of IPCD primarily for healthy or recreationally active individuals. The immediate increase in rSO_2_, reduction in tissue fluid content, and reduced fatigue levels indicate that IPCD combined with the supine position can be used for post-exercise muscle recovery as well as for managing fatigue accumulated during daily activities. Because significant improvements in rSO_2_ were observed within 10 min, IPCD in the supine position may be conveniently applied during short recovery intervals to enhance tissue oxygenation. However, it should be acknowledged that elite athletes may present distinct physiological adaptations compared to the healthy subjects in this study. Therefore, caution is warranted when generalizing these results to elite competitive levels, and further research is recommended to verify these effects in elite cohorts and to establish optimal application protocols.

This study has some limitations. First, the absence of a control group is a primary limitation. Consequently, while the rapid rSO_2_ changes likely reflect the effects of IPCD, the reductions in fluid content are likely attributable to both IPCD and supine positioning; thus, it is difficult to distinguish the specific contribution of IPCD from that of simply lying supine. Second, we did not measure the time-course changes in T2 values and subjective fatigue. Therefore, it remains uncertain whether a shorter intervention would have comparable effects on fluid reduction and fatigue relief. Third, because the preceding fatigue level was not standardized (e.g., via exercise or restricted activity), the rSO_2_ dynamics in supine position observed in this study may differ from physiological responses in fatigued muscles (Maia et al., 2024a). Fourth, although the INVOS system used in this study to evaluate rSO_2_ utilized spatially resolved spectroscopy to minimize superficial signal interference, and participants with thin subcutaneous tissues including fat were selected to ensure signal quality, NIRS signals could be influenced by cutaneous blood flow changes induced by compression (Buono et al., 2005). Thus, the rSO_2_ values may have partially reflected contributions from the cutaneous circulation. Finally, the pulse oximeter used for safety monitoring is prone to signal loss from artifacts; thus, local arterial occlusion could not be strictly evaluated. Based on these limitations, our findings should be interpreted with caution.

## 5 Conclusion

Our findings reveal that a 30-min IPCD intervention in supine position enhances tissue oxygen saturation and promotes fluid clearance in the muscle tissues of healthy individuals. The rSO_2_ levels plateaued within the first 10 min, while reductions in T2 values and lower leg circumference were observed after the 30-min intervention. These findings indicate that while oxygenation benefits occur early, a 30-min IPCD session is effective for providing both oxygenation and fluid reduction effects. Further studies are needed to determine whether shorter intervention durations can provide similar benefits and contribute to the development of more effective and time-efficient IPCD protocols for practical use.

## Data Availability

The raw data supporting the conclusions of this article will be made available by the authors.
