# Supplementary material for: Effects of intermittent pneumatic compression device on the improvement of tissue oxygen saturation and fluid clearance at the compression site
Source: Front Physiol. 2026 Feb 12;17:1725445. doi: 10.3389/fphys.2026.1725445 (PMC12935686; doi:10.3389/fphys.2026.1725445)
Supplement: Supplementary file 1 [file DataSheet1.docx]

Supplementary Material

# Supplementary Figures and Tables

## Supplementary Figures

**Supplementary Figure 1.** Intervention using an intermittent pneumatic compression device


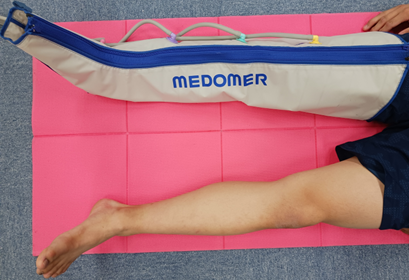


**Supplementary Figure 2.** Examples of rSO_2_ changes over time and measurement intervals


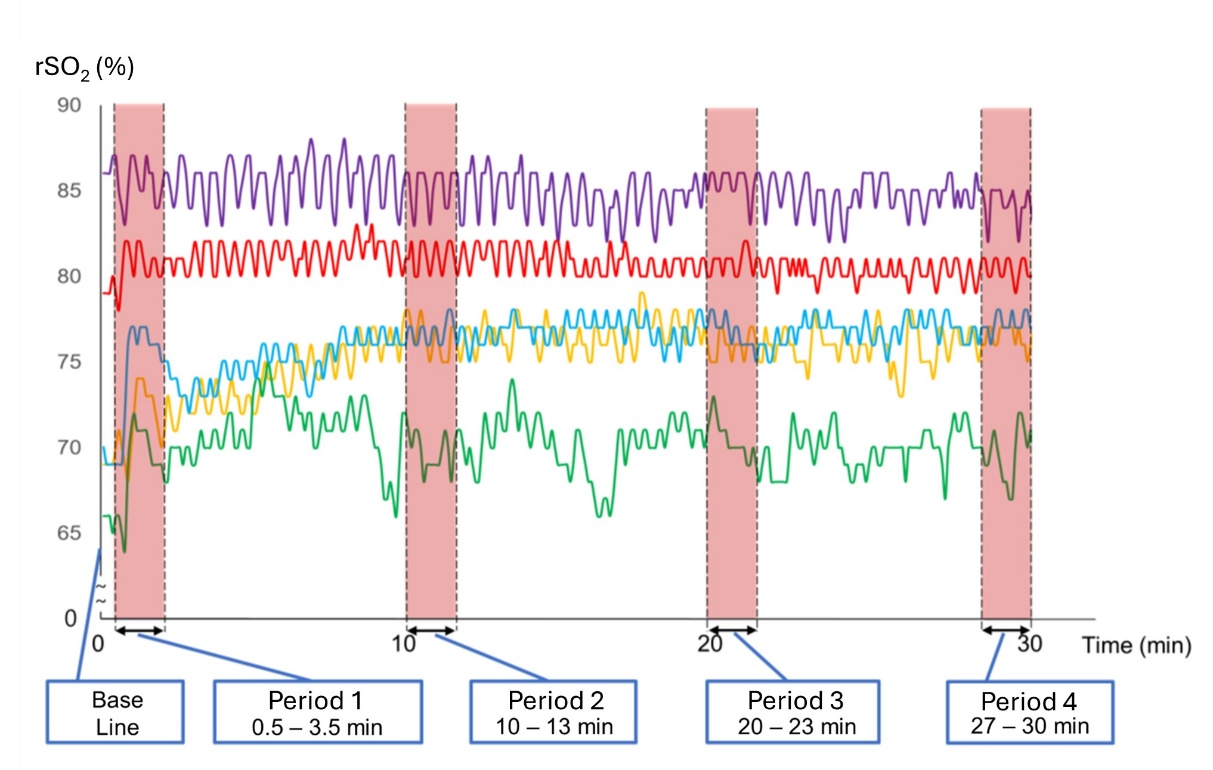


rSO_2_, regional oxygen saturation

## Supplementary Table

**Supplementary Table 1.** Difference in interference pressures with and without missed pulse wave detection

|  | Missed pulse wave  (n = 32) | No missed pulse wave  (n = 27) | p-value |
| --- | --- | --- | --- |
| Ankle [mmHg] | 87.0 ± 19.3 | 86.0 ± 23.5 | 0.86 |
| Leg [mmHg] | 81.1 ± 4.9 | 78.7 ± 7.3 | 0.16 |
| Popliteal [mmHg] | 58.9 ± 13.6 | 63.5 ± 14.2 | 0.23 |
| Thigh [mmHg] | 84.0 ± 3.6 | 82.4 ± 2.3 | 0.05 |

Unpaired t-test
